# ASSOCIATION OF RELIGIOUS ATTENDANCE, RELIGIOSITY/SPIRITUALITY, AND C-REACTIVE PROTEIN IN MIDDLE TO OLDER ADULTS IN THE U.S

**DOI:** 10.1093/geroni/igad104.1503

**Published:** 2023-12-21

**Authors:** Katherine Britt, Augustine Boateng, Joshua Sebu, Hayoung Oh, Ruby Lekwauwa, Lauren Massimo, Benjamin Doolittle

**Affiliations:** The University of Pennsylvania, Philadelphia, Pennsylvania, United States; The University of Pennsylvania, Philadelphia, Pennsylvania, United States; University of Cape Coast, Cape Coast, Western, Ghana; Georgetown University, Washington, District of Columbia, United States; Yale University School of Medicine, New Haven, Connecticut, United States; The University of Pennsylvania, Philadelphia, Pennsylvania, United States; Yale University School of Medicine, New Haven, Connecticut, United States

## Abstract

Elevated levels of C-reactive Protein (CRP), a biomarker of systemic inflammation, are associated with a higher risk of cognitive decline and dementia. Religious attendance and religiosity/spirituality are associated with better cognitive health, yet the underlying physiological mechanisms are not well understood. We examined the association between religious service attendance, religiosity/spirituality, and CRP levels in a nationally representative sample of U.S. adults aged 50 years and older (n=6,652) from the Health and Retirement Study from 2006 to 2014. The mean age was 70.7(9.32) years, 84.4% were White, 58.4% were female, and mean CRP levels were 3.98 (8.50) ug/mL. Utilizing the generalized estimating equation (GEE), a significant negative association was found between religious service attendance and religiosity/spirituality with average CRP levels, even after controlling for age, sex, education, marital status, race/ethnicity, income, body mass index (BMI), and health conditions (diabetes, cancer, hypertension) [B: -0.39, 95% CI:-0.76,-0.03, p<0.034 and B: -0.04, 95% CI:-0.07,-0.01, p<0.01]. Age, income, and education were negatively associated with CRP levels, while being female, Black/African American, having diabetes, cancer, hypertension, and BMI were positively associated with CRP levels. Findings contribute to our understanding of a possible mechanism by which religious service attendance and religiosity/spirituality influence cognitive health through reduced systemic inflammation. Interventions are needed to sustain religious attendance supporting religiosity/spirituality for adults who find religion/spirituality important, with the potential to reduce the risk of cognitive decline and dementia.

